# Miscellany

**Published:** 1888-11

**Authors:** 


### MISCELLANY.

### MEDICAL NOTES.

A general meeting of the Tōkyō Igaku Kwai (Tōkyō Medical Society) was held on May 24, at 6 p. m., in the room of physiology, University of Tōkyō, when the following addresses were given:

Dr. Hisayasu Mita—Influence of fevers on nervous diseases.

Dr. Yoshito Inoko—Experience of the medical value of the oil of gynocardea odorata and pterocarpus flavus.

Dr. Seiki Ogata—Experiments in preserving meat with carbonic acid.

Sixth general meeting of the Japanese Private Sanitary Society was held May 26, at 1 p. m., in the great hall of the Engineering College, at which about 1,000 members and guests were assembled, including both men and women.

The Obstetric and Gynæcological Society's first meeting was held June 2, at 3 p. m., when the following speeches were made:

Dr. Kamejiro Ishii—How to determine months of pregnancy.

Dr. Gen-iku Miyaki—Condition of fœtus born in the period of six months and twenty days.

Dr. Hēnoshin Inoue—Cerebral anæmia caused by abortion.

Dr. Kenzo Kusuda—Medical discrimination between multiparæ.

Dr. Ikujiro Sakurai—Artificial pregnancy.

Dr. Dekisuke Hanaoka brought a woman of 41 years old for diagnosis.

A meeting of the Society for Investigation of State Medicine was opened May 25, at 5.30 p. m., when the following speeches were made:

Dr. Kentaro Murata—How to cure syphilis.

Dr. Sakae Furukawa—Propagation of the people.

Promotion to rank and appointment to office—On 22d of May, Sankei Hagiwara was appointed to be physician to H. I. M. the Emperor.

On 29th, 2d class of merit, Kyokujitsu Jūkōshō, was conferred on Kensai Ikeda, Hakase, Physician to H. I. M. the Emperor and Director General of the Army, and Hōsei Ito, Physician to H. I. M. the Emperor.

On 7th, Degree of Hakase was conferred on each of the following persons: Wami Taguchi, Seiki Ogata, Masakichi Sasaki, Ryōsei Koganei, faculties of medicine, University of Tōkyō and Susumu Satō.

Examination for the Practice of Medicine at Tōkyō—The whole number of applicants was 1,989, of which 1,322 were examined and 667 were not examined, on account of sickness or other circumstances; of these, 593 passed successfully and the rest failed. Applicants for dentistry were seventeen, of whom five were successful and twelve failed.

Examinations for the same at Kyōtō—The whole number of applicants was 622, of whom 338 were examined and 284 not examined, on account of sickness or other circumstances, and 84 were successful; the rest failed.

Dismissal of Chinese Physicians from the charge of the Prince Haru-no-miya—As the treatment of Chinese physicians is generally mild, it is still thought by some Japanese, especially by old men and women, that children should be treated by them. Whether from this idea or not, the Prince Haru-no-miya has been heretofore under the care of the Chinese physicians Sōhaku Asada and Teiken Fukui. But recently they were dismissed, and now the prince is under the care of Kensai Ikeda, Physician to H. I. M. the Emperor.—*The Sei-i-kwai Medical Journal*, Tōkyō, Japan.

---

TRIBROMPHENOL AS AN ANTISEPTIC.—Grimm claims considerable antiseptic value for tribromphenol, which is separated by the action of bromine on carbolic water solutions in the form of soft, white crystals, fusible at 95 Cent. Tribromphenol, according to Grimm, does not irritate the mucous membrane of the mouth, pharynx, or nose; nor does it effect the skin, even near wounds. When the powder is sprinkled on recent wounds more or less violent burning ensues, and the wound surfaces are slightly cauterized, and become necrotic superficially. It severely irritates granulating wounds, and slight hæmorrhages sometimes occur. Atonic pale granulations are very quickly excited to reparative action by the drug. It acts as an energetic disinfectant on ulcers and gangrenous processes, and is, therefore, recommended for impregnating (2 to 3 per cent.) bandages, for antiseptic salves, preparations of bacilli, etc. It was found that a 1 per cent. ammoniacal solution destroyed the bacteria in putrefying liquids within thirty minutes. Trials of it with gauze saturated with various fermentable substances gave excellent results as showing its value as an antiferment and antiseptic.—*Deutsche Med. Zeitung*, No. 52, 1888.

---

HYPODERMIC INJECTIONS OF ANTIPYRIN.—Derlon claims that aqua lauro-cerasi is an excellent vehicle for a hypodermic injection of antipyrin, since it lessens the pain of the injection, though it adds to the pain of morphine solutions. Derlon uses the following formula :

Antipyrin .................. 30 grains.
Hydrochlor. of Cocaine..... 1 "
Aqua lauro-cerasi.......... 1 drachm.

*Revue Gen. de Clin. et de Therap.*, July 12, 1888.

---

USE OF ANTIPYRIN WITH QUININE.—Derlon says that if antipyrin is added to the prescription containing quinine in cases in which large doses of quinine are necessary (as much as 15 grains, or more), the unpleasant effects of the quinine are obviated. He adds 3 grains of antipyrin to each 5 grains of quinine. This, he says, also increases the antipyretic effect of the quinine, and prevents quinism. The mixture is better tolerated by the stomach than the quinine alone.—*Revue Gen. de Clin. et de Therap.*, July 15, 1888.

---

FORMULA FOR BRONCHITIS.—Céhron's formula is as follows :

R.
Terpine ............... 5 grammes.
Glycerine..............
Rectified spirits of wine..
Sirup of honey, aa......70 grammes.
Vanilline ...............2 centig.

S. Two tablespoonfuls daily.

Each teaspoonful contains 50 centig. (grs. 10) of terpine.

---

*University Medical Magazine.*—The first number of this *magazine*, edited under the auspices of the alumni and faculty of medicine of the University of Pennsylvania, has been issued, and in good style.

On the cover is a portrait of John Morgan, the founder of the medical faculty of the University of Pennsylvania. Also the names of the editorial staff, consisting of an advisory and an editorial committee. The table of contents embraces "Original Articles, Memoranda, Laboratory Notes, Department Notes, Editorial, Society Proceedings, Book Notices, Miscellaneous." The publisher is A. L. Hummel.

